# Potential role of *N*-acetyl glucosamine in *Aspergillus fumigatus-*assisted *Chlorella pyrenoidosa* harvesting

**DOI:** 10.1186/s13068-019-1519-3

**Published:** 2019-07-10

**Authors:** Arghya Bhattacharya, Megha Mathur, Pushpendar Kumar, Anushree Malik

**Affiliations:** Applied Microbiology Laboratory, Centre for Rural Development and Technology, Indian Institute of Technology, Delhi, Hauz Khas, New Delhi 110016 India

**Keywords:** Bioflocculation, *N*-acetyl glucosamine, Fungi, Algae, Atomic force microscopy

## Abstract

**Background:**

Algal harvesting is a major cost which increases biofuel production cost. Algal biofuels are widely studied as third-generation biofuel. However, they are yet not viable because of its high production cost which is majorly contributed by energy-intensive biomass harvesting techniques. Biological harvesting method like fungal-assisted harvesting of microalgae is highly efficient but poses a challenge due to its slow kinetics and poorly understood mechanism.

**Results:**

In this study, we investigate *Aspergillus fumigatus*–*Chlorella pyrenoidosa* attachment resulting in a harvesting efficiency of 90% within 4 h. To pinpoint the role of extracellular metabolite, several experiments were performed by eliminating the *C. pyrenoidosa* or *A. fumigatus* spent medium from the *C. pyrenoidosa*–*A. fumigatus* mixture. In the absence of *A. fumigatus* spent medium, the harvesting efficiency dropped to 20% compared to > 90% in the control, which was regained after addition of *A. fumigatus* spent medium. Different treatments of *A. fumigatus* spent medium showed drop in harvesting efficiency after periodate treatment (≤ 20%) and methanol–chloroform extraction (≤ 20%), indicating the role of sugar-like moiety. HR-LC–MS (high-resolution liquid chromatography–mass spectrometry) results confirmed the presence of *N*-acetyl-d-glucosamine (GlcNAc) and glucose in the spent medium. When GlcNAc was used as a replacement of *A. fumigatus* spent medium for harvesting studies, the harvesting process was significantly faster (*p* < 0.05) till 4 h compared to that with glucose. Further experiments indicated that metabolically active *A. fumigatus* produced GlcNAc from glucose. Concanavalin A staining and FTIR (Fourier transform infrared spectroscopy) analysis of *A. fumigatus* spent medium- as well as GlcNAc-incubated *C. pyrenoidosa* cells suggested the presence of GlcNAc on its cell surface indicated by dark red dots and GlcNAc-specific peaks, while no such characteristic dots or peaks were observed in normal *C. pyrenoidosa* cells. HR-TEM (High-resolution Transmission electron microscopy) showed the formation of serrated edges on the *C. pyrenoidosa* cell surface after treatment with *A. fumigatus* spent medium or GlcNAc, while Atomic force microscopy (AFM) showed an increase in roughness of the *C. pyrenoidosa* cells surface upon incubation with *A. fumigatus* spent medium.

**Conclusions:**

Results strongly suggest that GlcNAc present in *A. fumigatus* spent medium induces surface changes in *C. pyrenoidosa* cells that mediate the attachment to *A. fumigatus* hyphae. Thus, this study provides a better understanding of the *A. fumigatus*-assisted *C. pyrenoidosa* harvesting process.

**Electronic supplementary material:**

The online version of this article (10.1186/s13068-019-1519-3) contains supplementary material, which is available to authorized users.

## Background

Microalgae showcase diverse applications for wastewater treatment and subsequent bioenergy generation. Large quantity of microalgal biomass is the foremost requirement in the biofuel route, for which several high-end/advanced photobioreactors have already been designed by several researchers [1–3]. However, the commercialization of algal biofuels still lags behind due to high cost of investment towards energy-intensive biomass harvesting techniques like centrifugation, membrane filtration and chemical based flocculation from photobioreactors [4–6]. Chemical processes are highly efficient, but the requirement of a lot of flocculant dosages renders the harvested biomass contaminated with undesirable chemicals [7, 8]. Biologically induced harvesting of algal cells is now being explored as a replacement for the conventional algal dewatering processes which contribute towards 3–15% of the algal biomass production cost [9, 10]. The harvested biomass can be processed for biofuel generation without any loss of quality [11, 12]. There have been quite a few studies on the algal–algal and algal–bacterial interactions for bio-harvesting of algal cells from bulk media [13–20]. A very recent approach is the use of filamentous fungi for harvesting algal cells [21–23]. In spite of having the potential for becoming a cost-effective process for algal harvesting, the lack of knowledge regarding the causative factors for the algal attachment to fungal pellets limits its application.

When fungus is grown under submerged conditions with agitation, it forms a dense, compact hyphal structure termed as pellets [24]. When these pellet-forming filamentous fungi (PFF) were co-cultivated with algal cells, there is an orderly attachment of algal cells on the fungal pellets [22, 23, 25]. This phenomenon of algal–fungal attachment is of interest as it solves the problem of algal harvesting to a large extent. However, the studies with co-cultivation of fungi and algae have reported the interaction time to be between 24 and 72 h [22, 25–31]. In a more straight-forward approach, it has been observed that the attachment of algal cells (green algae or cyanobacteria) to fungal pellets could take place within 4–6 h using pre-cultivated fungal biomass [12, 32]. Such interaction of algae and PFF [12] is intriguing as it does not follow the simple kinetics of bio-harvesting [13, 33, 34]. Our recent study confirms that in addition to a specific set of physical conditions, the metabolically active fungus is mandatory for efficient attachment of the *Chlorella pyrenoidosa* cells to the *Aspergillus fumigatus* pellet [12]. This fact raises further queries on the biological context of *C. pyrenoidosa*–*A. fumigatus* attachment, which although seems far simpler than the complex algal–fungal associations in nature.

A complex symbiotic association between fungus and cyanobacteria exists in the form of lichens in natural systems [35]. In artificial systems, such as co-culturing of fungus (*Aspergillus nidulans*) with microalgae (*Chlamydomonas*), a mutualistic association for nutrient exchange has been reported as reflected by thinning of microalgal cell wall by enzymatic action of fungus. However, this mutualism may sometimes lead to antagonism, when the algal death occurs due to over secretions of these enzymes [36]. Relatively simple phytoplankton–parasitic fungal (Chytrids) interactions controlled by cell-to-cell contact also exist in nature, which are driven by chemotaxis [37]. In our recent studies, somewhat similar action of fungus was observed in algal–fungal pellets as fungus used algal cells as nutrient source by enzymatically degrading it [32]. However, what drives the interaction between *C. pyrenoidosa* and *A. fumigatus* at the molecular level is not clear. There are also no specific molecules reported for PFFs so far, although enough evidence for extracellular polymeric substances (EPS)-mediated aggregation exists [38–41].

The present study aims to find out the causative mechanism behind this interesting phenomenon. It also tries to answer questions like why a specific set of biological conditions is necessary for this process to occur. Experiments were designed to address several questions like is there any chemical signaling/mediating molecule which mediates the attachment of *C. pyrenoidosa* and *A. fumigatus,* what is the origin of this mediating molecule, i.e., *C. pyrenoidosa* or *A. fumigatus,* what is the type/nature of this mediating molecule, how does this molecule mediate the attachment, etc. However, this study raises further questions regarding the nature and type of receptors which may be responsible for the attachment process that needs to be investigated further.

## Results

### Role of extracellular metabolites in *C. pyrenoidosa*–*A. fumigatus* harvesting

**Fig. 1:**
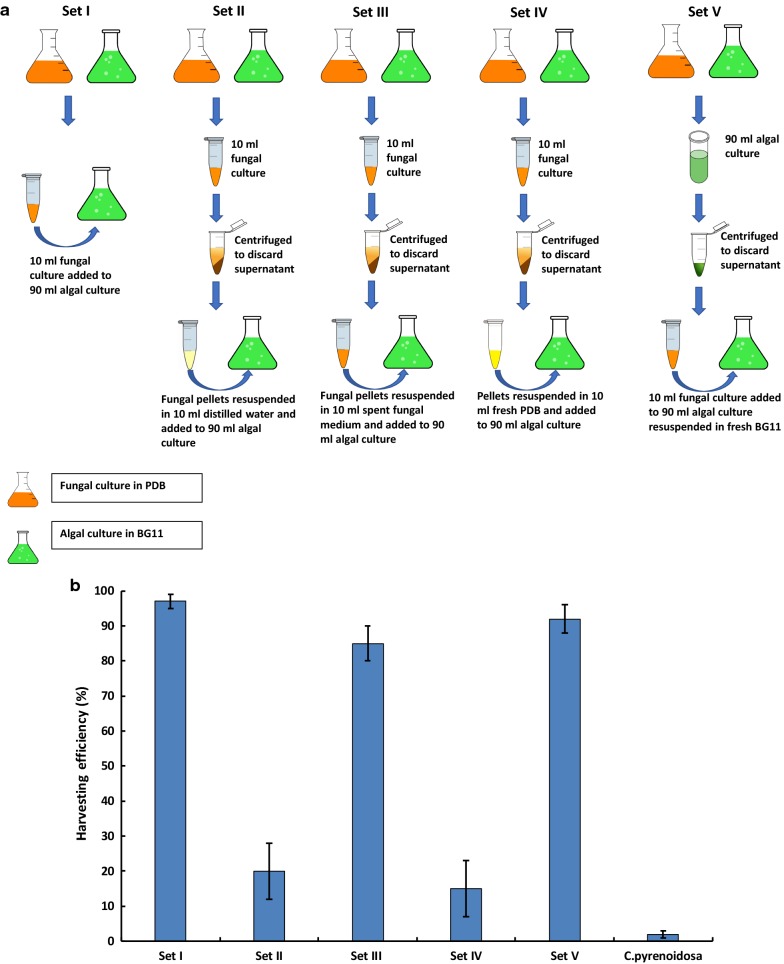
**a** Schematic representations of experimental conditions in different experimental sets. **b** Harvesting efficiency of BG11 grown *Chlorella pyrenoidosa* and *A. fumigatus* pellets as per different experimental conditions (defined in 1a) after 4 h (*n* = 3). Significant drop in harvesting efficiency is seen in Set II and Set IV (*p* < 0.05)

When *A. fumigatus* pellets and *C. pyrenoidosa* cells, suspended in respective spent medium (Fig. 1a), were mixed at 1:5 ratio, *C. pyrenoidosa* harvesting efficiency of 90% was observed after 4 h (Fig. 1b). This *A. fumigatus*–*C. pyrenoidosa* ratio and its harvesting efficiency were optimized in our earlier study [12] and has been referred to as control for all the experiments performed in the present study. To pinpoint the specific mediating molecule, i.e., whether the cell structure of *C. pyrenoidosa, A. fumigatus* or any of its extracellular metabolic by-product is mediating the attachment, several experiments were performed by eliminating the *C. pyrenoidosa* or *A. fumigatus* spent medium from the *C. pyrenoidosa*–*A. fumigatus* mixture. When unwashed *C. pyrenoidosa* cells were mixed with washed *A. fumigatus* pellets (Set II), the harvesting efficiency dropped to 20% compared to > 90% in the control (Set I). Interestingly, when washed *A. fumigatus* pellets were re-suspended in *A. fumigatus* spent medium (Set III), harvesting efficiency was similar to the control (Fig. 1b). However, when washed *A. fumigatus* pellets were resuspended in fresh PDB (Set IV), the harvesting efficiency was similar to that of washed *A. fumigatus* pellets, i.e., ≤ 20%. On the other hand, when washed *C. pyrenoidosa* cells were subjected to harvesting experiment with unwashed *A. fumigatus* pellets (Set V), > 90% harvesting efficiency was observed. *C. pyrenoidosa* culture without any *A. fumigatus* biomass showed ≤ 5% harvesting. Overall, significant reduction in harvesting efficiencies was seen (*p* < 0.05) when *A. fumigatus* spent medium was washed off. Therefore, impact of *A. fumigatus* spent medium on *C. pyrenoidosa* cells was explored further.

### Nature and characterization of the mediating molecule

**Fig. 2:**
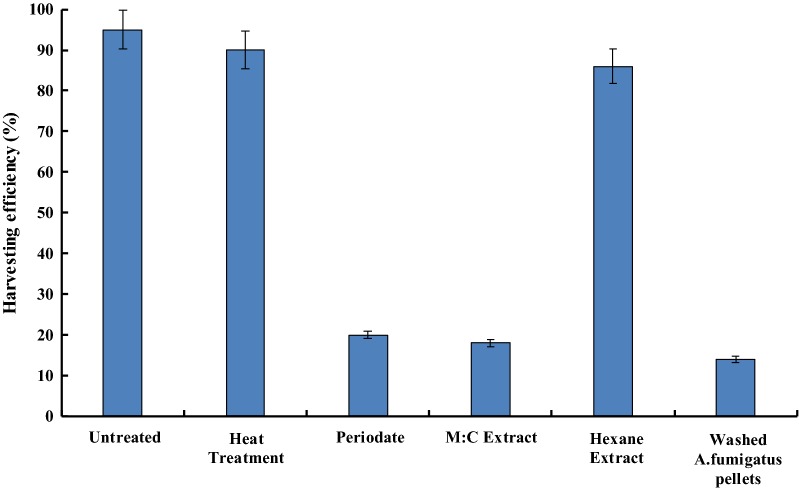
Harvesting efficiency of *C. pyrenoidosa* cells by *A. fumigatus* pellets after different treatments of *A. fumigatus* spent medium. Significant drop in flocculation efficiency is seen after periodate and methanol–chloroform treatment (*p* < 0.05) which is similar to the negative control (washed *A. fumigatus* pellets) without any *A. fumigatus* spent medium

To identify the nature of this extracellular molecule, different physical (heat) and chemical treatments (periodate, solvent extraction) of *A. fumigatus* spent medium were done before harvesting *C. pyrenoidosa* cells with untreated washed *A. fumigatus* pellets. The autoclaved *A. fumigatus* spent medium showed > 90% harvesting in 4 h. The aqueous phase of *A. fumigatus* spent medium after methanol: chloroform (1:1) extraction showed ≤ 20% harvesting efficiency. On the other hand, aqueous phase after hexane extraction exhibited > 90% harvesting efficiency. Periodate treatment of *A. fumigatus* spent medium leads to ≤ 20% harvesting efficiency. Harvesting efficiency of *C. pyrenoidosa* using *A. fumigatus* pellets with untreated spent was ≥ 90% (positive control). Harvesting efficiency of *C. pyrenoidosa* with washed *A. fumigatus* pellets (negative control) was ≤ 20% (Fig. 2).

The composition of *A. fumigatus* spent medium was studied using HR-LC–MS. The flow-through obtained using Strata-X CW column showed no loss of harvesting efficiency. Hence, the HR-LC–MS studies of this flow-through liquid were done. The results indicated the presence of two sugar-like molecules in the spent medium, i.e., *N*-acetyl-d-glucosamine (GlcNAc) and glucose (Table 1). HR-LC–MS of the supernatant of washed *A. fumigatus* pellets (without spent medium) did not show the presence of GlcNAc or glucose.

**Table 1:** Category of compounds obtained after HR-LC–MS of *A. fumigatus* spent media

Category of compound	Name of compounds
Sugars (Monosaccharides, Polysaccharides)	d-Glucose
Sugar derivatives (Sugar alcohols, ketones, aldehydes, esters)	*N*-Acetyl-d-glucosamine, 12a-hydroxy-5-deoxydehydromunduserone, Furo[3,4-b]pyridine-3-carboxylic acid, 5,7-dihydro-2-methyl-4-(2-nitrophenyl)-5-oxo-, methyl ester, acetyl tyrosine ethyl ester
Organic acids and its derivatives	l-2-Aminoadipic acid, 11-amino-undecanoic acid, phthalic acid mono-2-ethylhexyl ester
Hydrocarbons (Long chain and short chain)	3-Methylcholanthrene
Lipid, Fatty acids and its methyl esters	11-Amino-undecanoic acid, 6’-Hydroxysiphonaxanthin, *N*-ethyl arachidonoyl amine
Nitrates, Nitrites, amines and other nitrogen derivatives	Isoamyl nitrite
Protein metabolites (amino acid sequences)	Arg Arg Gln, Val Arg Gly, Ser Asn Gly, Leu Ala Arg
Vitamins and its derivatives	26,26,26,27,27,27-hexafluoro-1alpha, 24-dihydroxyvitamin D3, 1alpha, 25-dihydroxy-26,27- dimethyl-20,21,22,22,23,23-hexadehydro-24ahomovitamin D3, 2alpha-Fluoro-19-nor-22-oxa—1alpha, 25-dihydroxyvitamin D3, Phylloquinone (Vitamin K1)
Nucleic acids and its derivatives	Propylthiouracil glucuronide, Pseudouridine, 7,8-didehydroastaxanthin
Cytokines	Kinetin
Other metabolites and compounds	Zolpidem Metabolite I, dihydrodeoxystreptomycin

### Confirmation of the mediating molecule in *C. pyrenoidosa*–*A. fumigatus* interaction

Various treatments of *A. fumigatus* spent medium indicated sugar-like molecule to be responsible for harvesting. Hence, experiments were conducted to study the role of glucose and GlcNAc as a replacement of *A. fumigatus* spent medium during *C. pyrenoidosa*–*A. fumigatus* harvesting. Glucose, being the simplest form of sugar, was tested first. *C. pyrenoidosa* cells were incubated with 100-mM glucose and then harvesting experiments were carried out using washed *A. fumigatus* pellets devoid of any *A. fumigatus* spent medium. Results showed > 75% harvesting after 5 h, suggesting that glucose assisted in the harvesting process. Negative control of *C. pyrenoidosa* incubated without glucose did not show any harvesting with washed *A. fumigatus* pellets (Additional file 1: Figure S1).

**Fig. 3:**
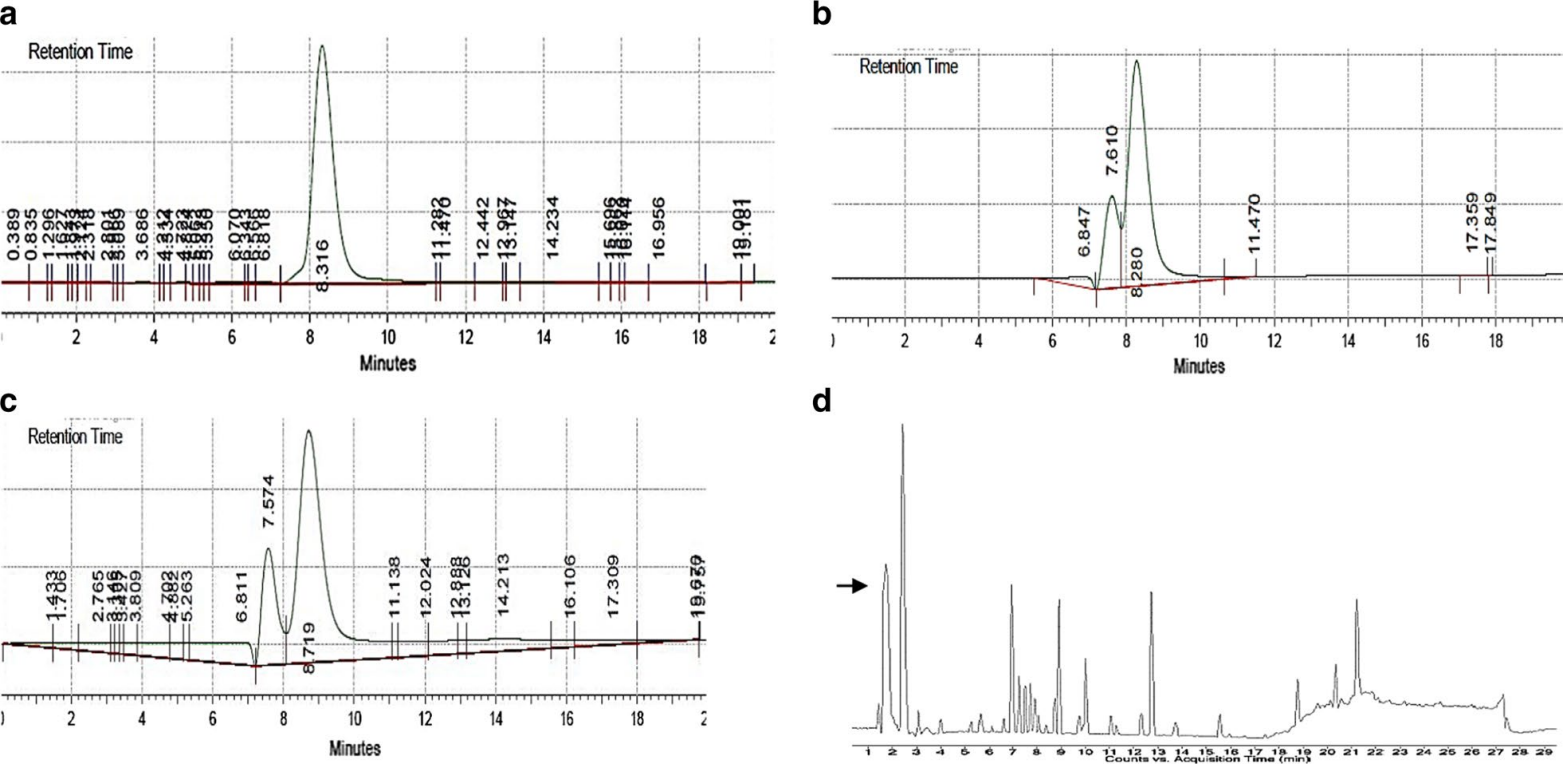
HPLC chromatograms of supernatant from glucose pre-incubated *C. pyrenoidosa* at different stages of incubation. **a** Initial glucose concentration (RT-8.3 min) of 94.36 mM. **b** Glucose concentration after 2 h of pre-incubation showing a reduced concentration up to 45 mM. **b** After harvesting, the peak shifted to RT-8.7 which corresponds to the peak of GlcNAc, depicting the formation of GlcNAc during the attachment process. **d** HR-LC–MS chromatogram of the supernatant after harvesting glucose pre-incubated *C. pyrenoidosa* and washed *A. fumigatus* pellets confirming the presence of GlcNAc (indicated by arrow)

The concentration of glucose in the supernatant at different stages of incubation and harvesting was analyzed using HPLC (Fig. 3). HPLC analysis showed the presence of glucose before and after incubation with *C. pyrenoidosa* cells. No other peak was observed in these spectra. However, after mixing with *A. fumigatus* pellets and harvesting for 5 h, no glucose was detected by HPLC, but 24 mM of GlcNAc was detected. This suggested that the conversion of glucose into GlcNAc took place during harvesting with *A. fumigatus* pellets. During HPLC, GlcNAc had a retention time of 8.7 min; while glucose had a retention time of 8.3 min. This was an interesting observation as after incubating *C. pyrenoidosa* with glucose, the end product after harvesting was not glucose but GlcNAc. HR-LC–MS analysis also confirmed the presence of GlcNAc after 5 h of harvesting the glucose-incubated *C. pyrenoidosa* cells (Fig. 3a–d). Therefore, the exact role of GlcNAc during harvesting was studied in further experiments. *C. pyrenoidosa* cells were incubated with 100-mM GlcNAc for 2 h and harvested with washed *A. fumigatus* pellets for 5 h. GlcNAc concentration was measured by HPLC during three stages of the experiments viz. initial, after 2-h incubation and after harvesting. The initial concentration of GlcNAc was 90.40 mM which decreased to 36 mM after incubation with *C. pyrenoidosa*. When the GlcNAc-incubated *C. pyrenoidosa* cells were subjected for harvesting with washed *A. fumigatus* pellets, harvesting efficiency was found to be > 85% and GlcNAc concentration was measured as 28 mM after 5 h. On the contrary, there was no GlcNAc detected in *C. pyrenoidosa* incubated without glucose/GlcNAc and subjected to harvesting with washed *A. fumigatus* pellets (Additional file 2: Table S1). No harvesting occurred in this case. However, when *t* test was performed between the harvesting efficiencies of *C. pyrenoidosa* cells incubated with glucose and GlcNAc, significant difference in harvesting efficiency was observed till 4 h. The difference was not significant after 4 h (Additional file 1: Figure S1).

### Microscopic analysis and surface characterization of *A. fumigatus* spent medium-/GlcNAc-incubated *C. pyrenoidosa* cells during harvesting

**Fig. 4:**
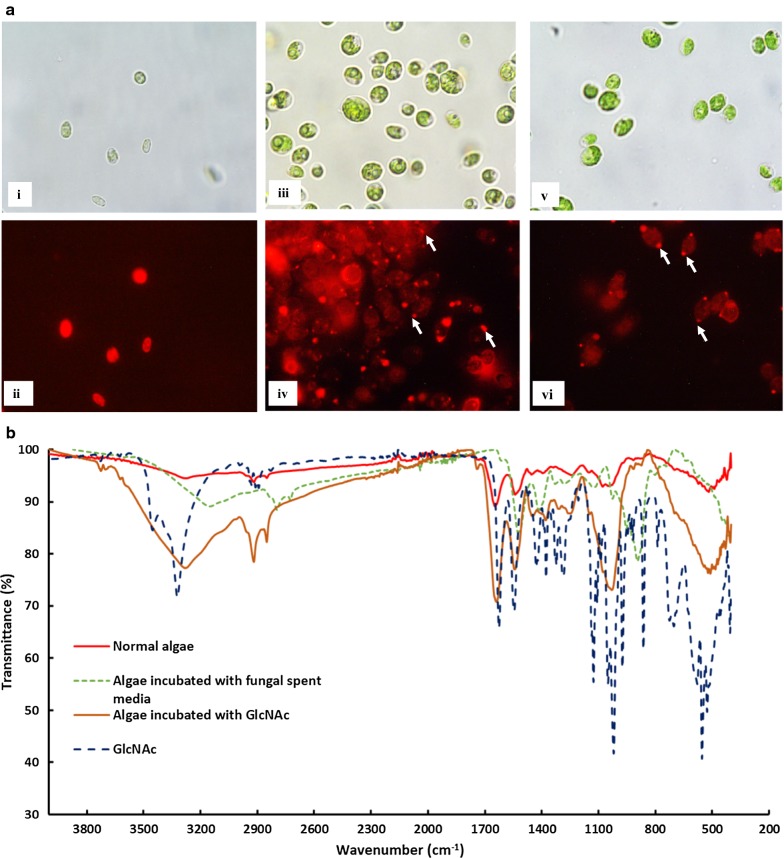
**a** Representative brightfield and fluorescent micrographs of Concanavalin A stained (i and ii) normal *C. pyrenoidosa*, (iii and iv) *C. pyrenoidosa* cells incubated with *A. fumigatus* spent medium and (v and vi) *C. pyrenoidosa* cells incubated with GlcNAc. White arrows indicate the presence of GlcNAc on the *C. pyrenoidosa* cell surface giving bright red spots. **b** FTIR spectra of normal *C. pyrenoidosa*, *A. fumigatus* spent media-incubated *C. pyrenoidosa*, GlcNAc-incubated *C. pyrenoidosa* and GlcNAc powder alone. Arrows indicate the characteristic peaks of GlcNAc in spent medium-incubated as well as GlcNAc-incubated *C. pyrenoidosa* biomass

HR-LC–MS and HPLC analyses suggested that washed *A. fumigatus* pellets could harvest *C. pyrenoidosa* cells in the presence of GlcNAc alone. This indicates that GlCNAc present in the *A. fumigatus* spent media is responsible for *C. pyrenoidosa* harvesting. To further investigate this observation, Con A staining of normal *C. pyrenoidosa* cells, *A. fumigatus* spent medium-incubated *C. pyrenoidosa* cells and GlcNAc-incubated *C. pyrenoidosa* cells was done. Results showed the absence of GlcNAc on the cell surface of normal cells. However, when the *C. pyrenoidosa* cells were incubated with *A. fumigatus* spent medium or GlcNAc, the presence of GlcNAc on its cell surface was indicated by dark red dots (Fig. 4a). Further, FTIR analysis of same samples was performed. The FTIR pattern of the *A. fumigatus* spent medium- and GlcNAc-incubated algae showed distinct peaks at 3430, 2922, 2131, 1657, 1564, 1380, 1319, 1161, 1079, and 894 cm^−1^. These peaks were found to be characteristic of GlcNAc when correlated with FTIR spectra of GlcNAc alone. No such peaks were observed in normal *C. pyrenoidosa* cells. The FTIR of *C. pyrenoidosa* alone showed the presence of phosphate (1048 cm^−1^), amide (1541 cm^−1^), carboxylic (1650 cm^−1^), alkenes (2919 cm^−1^) and hydroxyl (3300 cm^−1^) groups on its cell surface (Fig. 4b) [12].

**Fig. 5:**
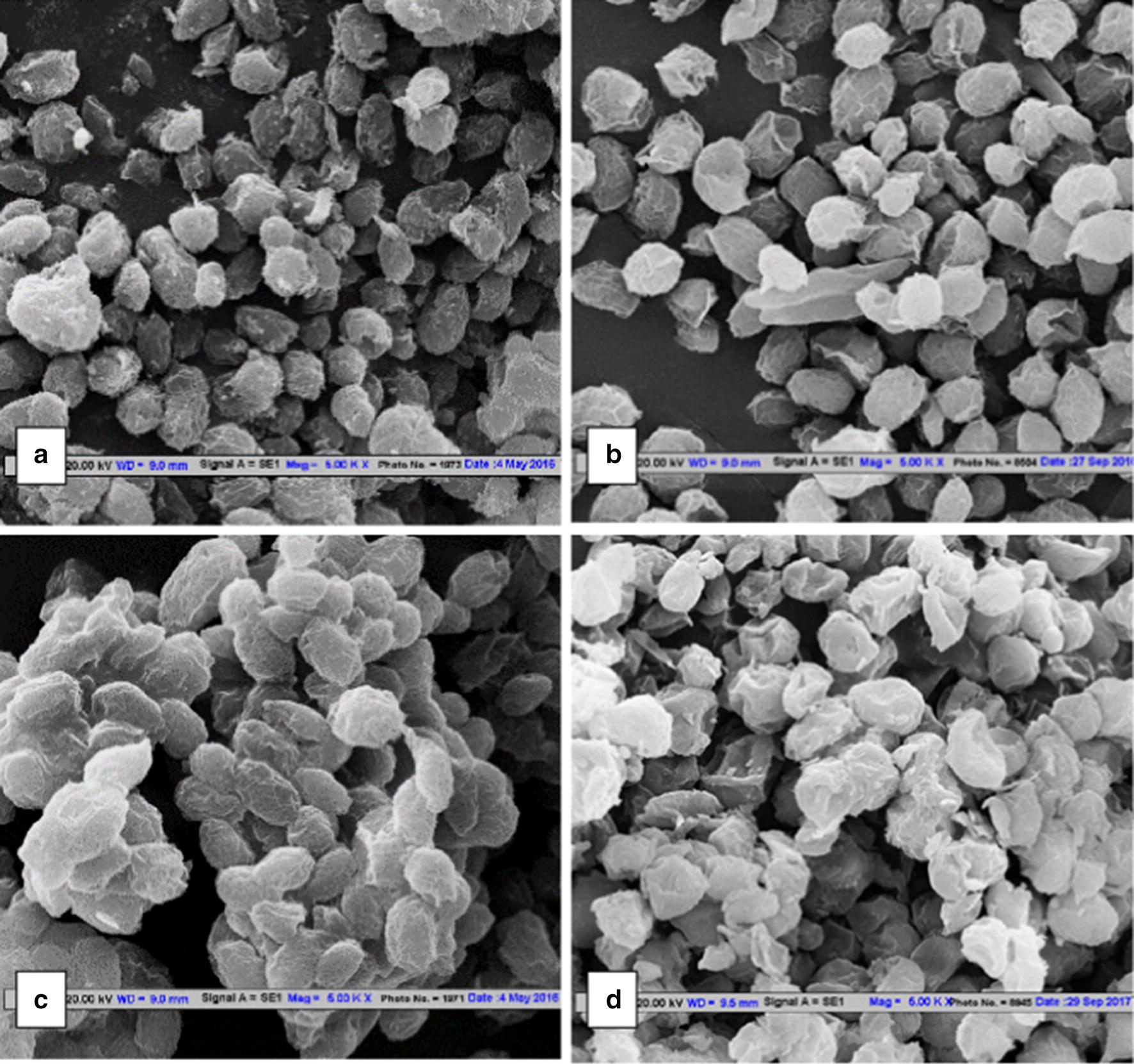
Scanning electron micrographs of (**a**) normal *C. pyrenoidosa* (**b**) *C. pyrenoidosa* incubated with washed *A. fumigatus* pellets (2.5 h) (**c**) *C. pyrenoidosa* incubated with *A. fumigatus* spent medium (2.5 h) (**d**) *C. pyrenoidosa* incubated with GlcNAc (2.5 h). The images shown above are the representative images for each treatment selected out of multiple frames. The figure shows the change in surface morphology of *C. pyrenoidosa* cells after incubating with *A. fumigatus* spent medium and GlcNAc while no such change in *C. pyrenoidosa* cells incubated with washed *A. fumigatus* pellets (2.5 h)

A distinct change in surface nature of *C. pyrenoidosa* cells incubated with *A. fumigatus* spent medium as well as GlcNAc for 2.5 h was observed through microscopic analysis (SEM and HR-TEM). The SEM image clearly showed more wrinkles on the *C. pyrenoidosa* cells after incubation as compared to the normal un-incubated *C. pyrenoidosa* cells (Fig. 5). The spent medium-incubated *C. pyrenoidosa* cells were elongated compared to the normal *C. pyrenoidosa* cells and appeared to be embedded in a matrix. Similar changes were observed when *C. pyrenoidosa* cells were incubated with GlcNAc. On the other hand, the SEM of the *C. pyrenoidosa* cells incubated with washed *A. fumigatus* pellets (which did not induce attachment) was found to be similar to the normal *C. pyrenoidosa* cells (Fig. 5).

**Fig. 6:**
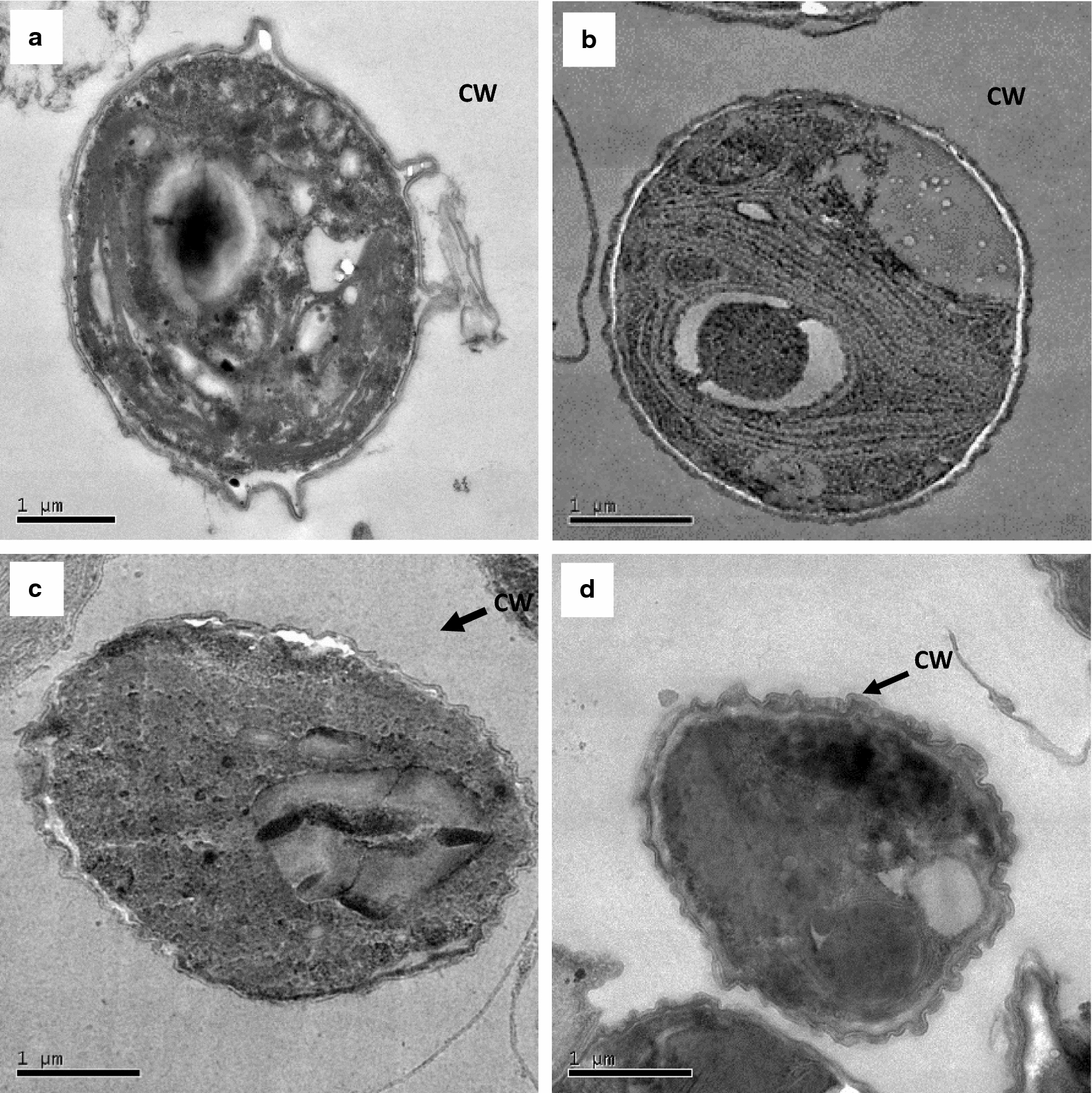
Transmission electron micrographs of (**a**) normal *C. pyrenoidosa* cells (**b**) *C. pyrenoidosa* incubated with washed *A. fumigatus* pellets (2.5 h) (**c**) *C. pyrenoidosa* incubated with *A. fumigatus* spent medium (2.5 h) (**d**) *C. pyrenoidosa* incubated with GlcNAc (2.5 h); CW denotes the cell wall of algal cells. The arrows show the formation of villi-like structures on the cell wall after incubation with *A. fumigatus* spent medium/GlcNAc. The images shown above are the representative images for each treatment selected out of multiple frames

To study the ultrastructural changes, HR-TEM of the above samples was performed. The normal *C. pyrenoidosa* cells and cell incubated with washed *A. fumigatus* (without *A. fumigatus* spent medium/GlcNAc) showed intact and round cells having smooth cell wall and well-defined cell organelles (Fig. 6). TEM images of *A. fumigatus* spent medium- and GlcNAc-incubated *C. pyrenoidosa* cells showed identical morphological changes. Both the images showed the formation of small villi-like structures on the cell surface. However, the TEM images of *C. pyrenoidosa* cells incubated with washed *A. fumigatus* pellets did not show such morphological changes and were similar to that of normal *C. pyrenoidosa* cells. The changes in the *C. pyrenoidosa* surface after *A. fumigatus* spent medium/GlcNAc treatment or even after complete harvesting does not affect the integrity of the cell wall as depicted by SYTOX green staining. The red auto-fluorescence was observed in *A. fumigatus* spent medium-incubated *C. pyrenoidosa* cells indicating live cells. As SYTOX binds to nucleic acids, dead cells would have shown green fluorescent color (Additional file 3: Figure S2).

**Fig. 7:**
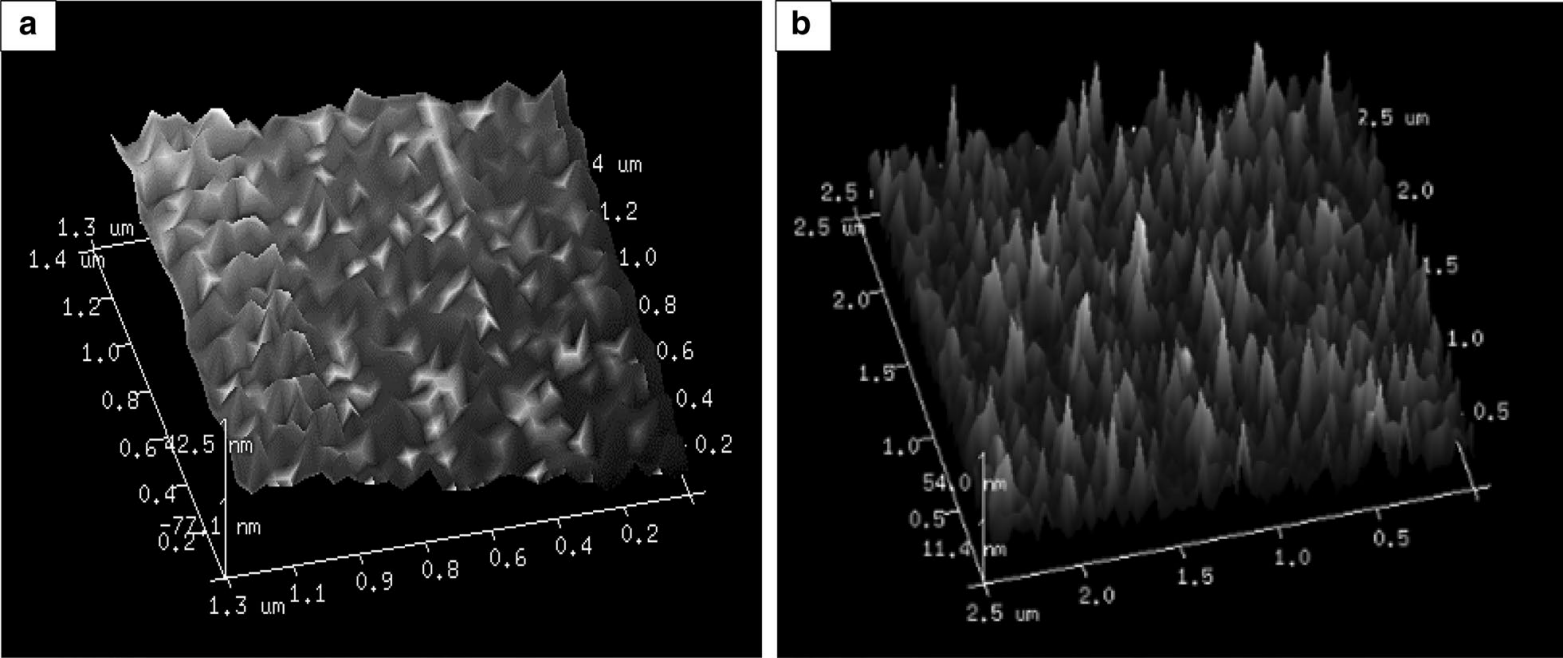
AFM analysis of (**a**) normal *C. pyrenoidosa* (RMS 76.5 nm) (**b**) spent medium treated *C. pyrenoidosa* (91.0 nm) showing change in the roughness of the cells after incubation with *A. fumigatus* spent medium

To confirm the surface changes observed through SEM and HR-TEM, AFM analysis of normal *C. pyrenoidosa* cells and *A. fumigatus* spent medium-incubated *C. pyrenoidosa* cells was conducted. Increase in the roughness of the *C. pyrenoidosa* cells (RMS value of 91.0 nm) was seen when incubated with *A. fumigatus* spent medium as compared to the normal *C. pyrenoidosa* which showed RMS value of 76.5 nm (Fig. 7). The cell height also increased from 42.5 nm in normal *C. pyrenoidosa* to 53.8 nm in the *A. fumigatus* spent medium-incubated *C. pyrenoidosa*.

## Discussion

Chemical harvesting, although quick, contaminates the biomass with unnecessary chemical contaminants [42]. Previous reports have suggested that biological harvesting of micro-algae improves the quality of the biomass for biofuel purpose. Wrede et al. [30] reported that there is a significant increase in lipid yield when microalgal cells were harvested with *A. fumigatus*. Increase in lipid content for algal–fungal pellets was also reported by Bhattacharya et al. [12]. In another study, *Botryococcus braunii* was harvested using *A. fumigatus* and the resultant biomass did not show any significant variation in the biomass composition. Apart from biodiesel aspect, harvesting of microalgae using filamentous fungi also increases its biogas potential. Prajapati et al. [32] reported enhanced bio-methane production from algal–fungal pellets due to the enzymatic degradation of microalgal cell wall by the fungi. All these studies show that harvesting algae with filamentous fungi is a favorable process for biofuel production. However, as the underlying mechanism of the process is not clearly understood, it is difficult to replicate the process at large scale. This study gives an insight into the communication mechanism between *A. fumigatus* and *C. pyrenoidosa* which could be exploited for fungal-assisted microalgal harvesting processes at large.

Chemical communication between microbial cells is an inherent property which dictates the attachment and aggregation of cells [41, 43]. Such communications mediated by secretion of extracellular chemical signaling molecules could impose inter-beneficial partnerships for both the species [44]. In natural systems, the algae and fungus exhibit a host–parasite relationship as seen in chytrid–phytoplankton association [37]. In artificial systems, the co-culturing of *Chlamydomonas reinhardtii* and *A. nidulans* strains has been reported to show obligate mutualism in terms of nitrogen and carbon exchange within the species. The fungus converts glucose into CO_2_, which is being used by algae for its growth. On the other hand, the nitrogen fixing *Chlamydomonas* sp. reduces nitrite to ammonia, which fungus can use as a nitrogen source [36, 45]. During this nutrient exchange, the cell wall of *Chlamydomonas* sp. attached to fungus was reported to show thinning due to the action of fungal remodeling enzymes [45].

The present study can be closely related to the above-mentioned cases as it also reports extracellular secretions from *A. fumigatus*, mediating cell wall changes in the *C. pyrenoidosa* cells. These secretions might help *A. fumigatus* to provide signal to the *C. pyrenoidosa* cell and bring it into close vicinity for nutritional benefits or enzymatic degradation [32]. Similar phenomenon of microalgal–fungal attachment has been reported by our group with wide range of microalgal species encompassing green algae (*Chlorella* sp.), blue–green algae (*Chroococcus* sp.) as well as mixed consortia [12, 32].

Fungi produce various exopolysaccharides during its growth [38], which have been known to mediate cell–cell adhesion. The present study has identified an extracellular causative factor produced by the fungus *A. fumigatus* which is responsible for the *C. pyrenoidosa*–*A. fumigatus* attachment. Experiments done with washed and unwashed *A. fumigatus* pellets have shown the presence of a mediating molecule in the *A. fumigatus* spent medium that is crucial for the attachment process. Washed *A. fumigatus* pellets could not induce attachment since they were devoid of any *A. fumigatus* spent medium. Fresh PDB also did not have the mediating molecule, and its harvesting efficiency harvesting was similar to the washed *A. fumigatus* pellets. This observation suggested that the causative factor is the extracellular metabolite produced by actively growing fungi. The result was also in agreement with our recent observations that relatively old (72-h grown) or autoclaved *A. fumigatus* pellets failed to harvest the *C. pyrenoidosa* cells due to their low metabolic activity and damaged hyphae [12]. *A. fumigatus* spent medium is a cocktail of extracellular metabolites including EPS which is a complex mixture of polysaccharides, proteins, nucleic acids and amyloids [40]. EPS production by *Aspergillus* sp. is reported for bio-flocculation [46]. However, to pin-point the causative factor, it is necessary to know the nature of the mediating molecule.

The autoclaving of the *A. fumigatus* spent medium did not affect the harvesting efficiency of the fungus. This indicated that the mediating factor was not proteinaceous in nature as autoclaving would denature the protein. Chloroform and methanol mixture was able to extract the polar organic components from the *A. fumigatus* spent medium. Loss of activity after this treatment indicated that the mediating factor was a polar organic compound. This was further confirmed using hexane as a solvent for extraction. Treatment of the *A. fumigatus* spent medium with hexane did not show any loss in harvesting activity. As hexane is able to extract only non-polar organic compounds, it further confirmed that the molecule is a polar organic compound. Further insights into the nature of the molecule were obtained by treating the *A. fumigatus* spent medium with sodium per-iodate. Per-iodate targets any saccharide/polysaccharide leading to its oxidation. Loss of harvesting activity after per-iodate treatment of the *A. fumigatus* spent medium strongly suggested that the mediating factor was a sugar-like molecule. The HR-LC–MS analysis of the *A. fumigatus* spent medium also showed the presence of two sugars, glucose and GlcNAc which might be the triggering factors for the attachment process. Previously, the bioflocculant produced by *Achromobacter xylosoxidans* was found to be composed mainly of carbohydrate hetero polymer [47]. In another study, characterization of a bioflocculant produced by *Aspergillus flavus* showed that it contained 69.7% sugar of which 1.8% was amino sugar-like GlcNAc [46]. In a recent study, it has been found that an amino sugar galactosaminogalactan mediates attachment of *A. fumigatus* to epithelial cells [48].

In the present study, we were able to pinpoint the particular molecule that could replicate the *C. pyrenoidosa*–*A. fumigatus* attachment process in the absence of *A. fumigatus* spent medium. Results of several spent treatments corroborated with HR-LC–MS results suggested the target molecule to be glucose or GlcNAc. The presence of either GlcNAc or *A. fumigatus* spent medium is mandatory for the attachment process. The *t* test between harvesting kinetics of control and GlcNAc-incubated *C. pyrenoidosa* cells did not show any significant difference. When GlcNAc was used for harvesting studies, it was clearly seen that the harvesting process was significantly faster as compared to glucose. HR-LC–MS and HPLC results confirmed that glucose is converted into GlcNAc by *A. fumigatus* pellets. Conversions of sugar-like molecules into GlcNAc have been reported by saprophytic fungus using degrading/hydrolytic enzymes [49, 50]. Kinetics of glucosamine formation using glucose and other sugars as substrate by fungus like *Aspergillus* sp. has also been reported during submerged fermentation [51]. Similar harvesting experiments with glucose anomers (Galactose and Mannose) showed only 30% harvesting which further suggests the role of GlcNAc in the harvesting process since these glucose anomers cannot be converted into GlcNAc (Additional file 4: Figure S3). The present result explains the previously observed pre-requisites for *C. pyrenoidosa*–*A. fumigatus* attachment (the presence of metabolically active fungi, temperature of 38 °C and neutral pH) [12], which seem to favor GlcNAc production. Earlier report showed that GlcNAc production by *Aspergillus* sp. was dependent on the pH of the system, where higher pH inhibited its formation [51]. Comparative study of GlcNAc production among three wild-type fungi viz*. A. fumigatus*, *Rhizopus oligosporus* and *Monascus pilosus* showed that *A. fumigatus* had the highest production capacity among the three fungi.

We could demonstrate the presence of GlcNAc on the spent medium-incubated and GlcNAc-incubated *C. pyrenoidosa* using the lectin Concanavalin A (Con A) stain, and FTIR. Although, Con A gives signal for glucose as well as glucosamine, *C. pyrenoidosa* suspended in BG11 only do not show any signal as seen in the Fig. 4a. When *C. pyrenoidosa* is incubated with *A. fumigatus* spent media or glucosamine, Con A shows signals on the algal cell surface (Fig. 4b, c). When *C. pyrenoidosa* cells were incubated in the presence of glucosamine alone, the concentration of glucosamine decreases over time. The FTIR pattern of glucosamine-incubated cells show peaks similar to glucosamine. The Con A staining results complement the findings of the FTIR data. The mechanism of interaction could be further elaborated by examining the effect of *A. fumigatus* spent medium on *C. pyrenoidosa* cell’s morphology, ultrastructure and cell surface roughness. Roughness analysis is one of the parameters which indicates the changes in the cell surface due to changes in the growing environment. The root mean square (rms) value corresponds to the roughness of the sample. AFM analysis showed that the roughness of the *A. fumigatus* spent medium-incubated *C. pyrenoidosa* cells was increased compared to the normal *C. pyrenoidosa* cells. Increase in roughness is an essential step for the cellular attachment process [52]. It has also been reported that in case of a green fouling alga *Enteromorpha*, increased roughness of cells caused more fouling compared to smooth cells with low roughness [53]. SEM micrographs (Fig. 5c) of spent medium-incubated cells depicted elongated *C. pyrenoidosa* cells embedded in a sticky matrix. Extracellular release of mucilage for entrapment is well documented for parasitic type of *A. fumigatus* species [40].

The fact that both *A. fumigatus* spent medium-incubated *C. pyrenoidosa* and GlcNAc-incubated algae showed the presence of GlcNAc-specific peaks in the FTIR spectra suggests that similar cell surface changes may be induced by *A. fumigatus* spent medium and GlcNAc. In this connection, SEM micrographs of GlcNAc-incubated *C. pyrenoidosa* cells also showed elongation and wrinkles on the cell surface. TEM images clearly show that *A. fumigatus* spent medium induces the formation of villi-like structures on *C. pyrenoidosa* surface. It was interesting to note that similar villi-like structures were induced by incubating *C. pyrenoidosa* cells with GlcNAc. On the other hand, washed *A. fumigatus* pellets (without *A. fumigatus* spent medium) failed to induce such changes. The above results strongly suggest that GlcNAc present in *A. fumigatus* spent medium is responsible for inducing surface changes in *C. pyrenoidosa* cells that mediates the attachment to *A. fumigatus* hyphae .

**Fig. 8:**
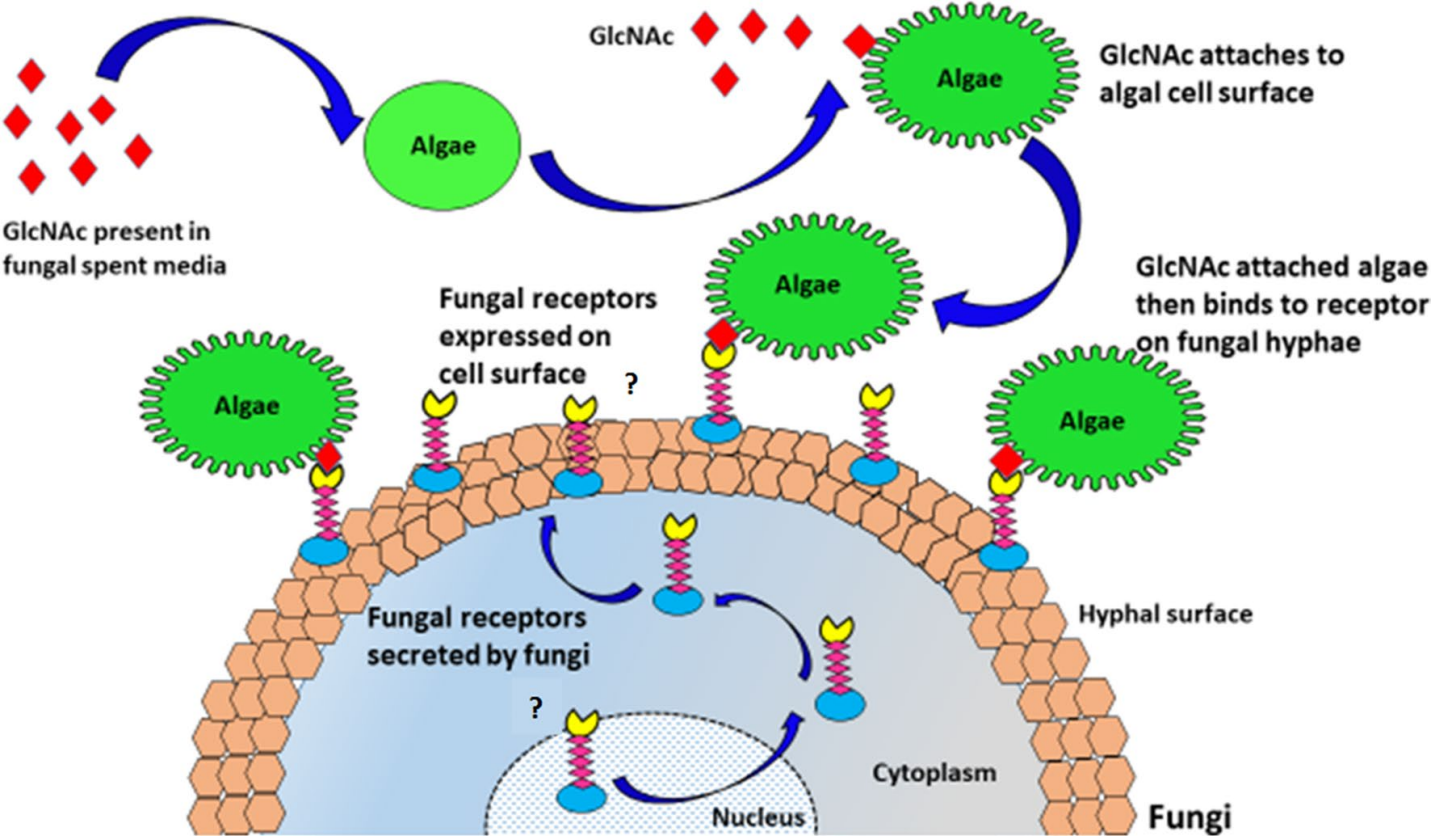
The figure depicts the probable mechanism of *C. pyrenoidosa*–*A. fumigatus* interaction mediated by GlcNAc (mediating molecule)

Role of GlcNAc has also been demonstrated for cell–cell adhesion in bacteria and yeast [54, 55]. Moreover, it has also been established that GlcNAc can function as a signaling molecule or as an inducer of hyphal growth in *Candida albicans* without undergoing a metabolic reaction [56]. It is also known to induce formation of curly fibers in bacteria [57]. In the present study, we demonstrate that GlcNAc plays a similar role in adhesion of *C. pyrenoidosa* cells to *A. fumigatus* pellets by inducing morphological changes on the *C. pyrenoidosa* cell surface which is not reported till now. In summary, we show that the *C. pyrenoidosa* attachment to *A. fumigatus* pellets is mediated by GlcNAc as depicted by biochemical and analytical methods. Based on the results, the probable mechanism of the process is depicted in Fig. 8. When the *C. pyrenoidosa* cells are incubated with *A. fumigatus* spent medium or GlcNAc, it attaches with the *C. pyrenoidosa* surface. This induces surface modifications in *C. pyrenoidosa* cells as the cells become elongated with development of projections on the surface. The GlcNAc molecule on *C. pyrenoidosa* might act as a chemical signal for *A. fumigatus* receptors, thereby attaching the *C. pyrenoidosa* cells onto *A. fumigatus* cell wall. Recent reports have shown the role of G protein-coupled receptors (G-PCRs) present in ascomycetes fungi for sensing sugars [58]. Also, it has been reported that the receptors on the *A. fumigatus* cell surface are dynamic and are made only in the presence of external stimuli [59]. This kind of receptors might play a role in the *C. pyrenoidosa*–*A. fumigatus* attachment for which metabolically active fungi are required. Our results indicate that this attachment is triggered by GlcNAc which may act like a quorum-sensing molecule. GlcNAc induces morphological changes on the *C. pyrenoidosa* cell surface which is clearly visible by TEM micrograph. However, our recent study with such microalgal–fungal attachment has shown that fungus uses this interaction process to ultimately utilize the microalgal cells as source of food [32]. Hence, this microbial interaction is different from other interactions where the emitter and receiver of the signal are both benefited [44]. The existence of *A. fumigatus* receptors and factors triggering their expression needs to be investigated further.

## Methods

### Organism and culture conditions

Microalgal species *Chlorella pyrenoidosa* was obtained from National Collection of Industrial Microorganisms (NCIM), NCL Pune (India). *C. pyrenoidosa* was maintained in 2% algae culture agar (HiMedia M343-500G) slants supplemented with BG11 (HiMedia, M1541-500G) in a plant growth chamber (Daihan Labtech, LGC-5101). Liquid cultures were maintained in BG11 broth at 120 rpm without CO_2_ supplementation. For experimental purposes, *C. pyrenoidosa* culture was grown in 2.5-L flasks under continuous light in a greenhouse maintained at 25 ± 1 °C and a light intensity of ≈ 3500 Lx [12]. The fungal strain *Aspergillus fumigatus* (Accession no. KY241789), previously isolated from wastewater [60], was used as the harvesting organism. *A. fumigatus* was maintained on sterile potato dextrose agar (PDA) slants (Himedia M096-500G) at 28 ± 1 °C.

### *C. pyrenoidosa* and *A. fumigatus* harvesting experiments

*Aspergillus fumigatus* slant (3 days old) was used to inoculate potato dextrose broth (PDB; Himedia M403-500G) medium for cultivation. *A. fumigatus* spore suspension prepared with 0.1% Tween-80 (≈ 10^8^ spores ml^−1^) was inoculated in 100-ml potato dextrose broth and incubated for 24 h at 28 ± 1 °C and 150 rpm in an orbital shaker. *C. pyrenoidosa* cultures exhibiting optical density (OD_680_) ≈ 2.5 were used for harvesting experiments. Ten milliliters of overnight-grown *A. fumigatus* culture was added to 90 ml of *C. pyrenoidosa* culture having OD_680_ ≈ 2.5 (1:5 dry weight basis) and kept at 38 ± 1 °C and 100 rpm for 4 h in an incubator shaker. The harvesting efficiency based on the optical density of *C. pyrenoidosa* was measured after every 30 min, for which the flask was allowed to stand for 3 min followed by drawing of the sample for absorbance measurement (OD_680_) using a microplate reader (Biotek EON^®^-C). Harvesting efficiency (HE) was calculated as:

$${\text{HE}}\, = \,\left( {1\, - \,\frac{{{\text{OD}}_{t} }}{{{\text{OD}}_{0} }}} \right)\, \times \, 100,$$

where OD_*t*_ = optical density at time *t* and OD_0_ = initial optical density.

To check the viability of normal *C. pyrenoidosa* cells and *C. pyrenoidosa* cells incubated with *A. fumigatus* spent medium during harvesting (2.5 h), the cells were stained with a nucleic acid stain, i.e., SYTOX green (Cat. No. S7020, Invitrogen), which specifically stains the dead or damaged cells. Following this, dual fluorescence was used to show red autofluorescence for live *C. pyrenoidosa* cells and green fluorescence of SYTOX green (Excitation/Emission: 504/523 nm) for dead cells [61].

### Role of extracellular factors

To assess the role of an extracellular substance for the attachment process, *A. fumigatus* biomass was washed twice with distilled water removing all the spent medium from it. The *A. fumigatus* medium present after the growth of the fungus has been termed as *A. fumigatus* spent medium throughout the text. The *A. fumigatus* biomass was subjected to 5 harvesting experimental sets viz: Set I—Control (unwashed *C. pyrenoidosa* and *A. fumigatus* pellets), Set II—Unwashed *C. pyrenoidosa* and washed *A. fumigatus* pellets, Set III—Unwashed *C. pyrenoidosa* and washed *A. fumigatus* pellets resuspended in its spent medium, Set IV—Unwashed *C. pyrenoidosa* and washed *A. fumigatus* pellets resuspended in fresh PDB, Set V—Washed *C. pyrenoidosa* and unwashed *A. fumigatus* pellets. Harvesting of all the above sets was performed similarly as described above for 4 h. Experiments were done in triplicates for each and every experimental set. *C. pyrenoidosa* culture without any *A. fumigatus* biomass was run as a negative control to check the sedimentation rate of *C. pyrenoidosa* culture. The experimental setup is graphically shown in Fig. 1a.

To study the effect of an extracellular factor (present in *A. fumigatus* spent medium) on the *C. pyrenoidosa* cell surface, scanning electron microscopy (SEM) and High-Resolution Transmission electron microscopy (HR-TEM) were performed before harvesting (normal *C. pyrenoidosa* cells) and after designated time during harvesting (2.5 h). For comparison, the SEM and HR-TEM of *C. pyrenoidosa* cells incubated with washed *A. fumigatus* pellets (without *A. fumigatus* spent medium) from Set II were also performed. The atomic force microscopy (AFM) of normal *C. pyrenoidosa* cells and cells during harvesting was performed to evaluate the change in cell height and roughness.

### Treatment and characterization of *A. fumigatus* spent medium

By the above preliminary experiments, the role of some extracellular factors presents in the *A. fumigatus* spent medium was highlighted. Hence, various treatments of *A. fumigatus* spent medium like autoclaving, sodium periodate treatment, Methanol: Chloroform extraction and hexane extraction were performed followed by testing the harvesting efficiency of these pre-treated *A. fumigatus* spent medium when added to washed *A. fumigatus* pellets and *C. pyrenoidosa* cells. For every experiment, *A. fumigatus* pellets were first washed to remove any spent medium present. Harvesting experiments were done with normal *C. pyrenoidosa* cells and *A. fumigatus* pellets resuspended in fungal spent medium after treatments (autoclaving, sodium periodate treatment, Methanol: Chloroform extraction and hexane extraction). A mixture of *C. pyrenoidosa* cells and *A. fumigatus* pellets with untreated spent was used as a positive control. A negative control comprising *C. pyrenoidosa* mixed with washed *A. fumigatus* pellets was run to ensure that the pellets of *A. fumigatus* did not have the fungal spent media.

*Aspergillus fumigatus* spent medium was autoclaved for 15 min at 121 °C and 15 psi pressure before using for harvesting experiments. As autoclaving would denature any protein component in the spent *A. fumigatus* medium, the role of protein molecule (if any) could be found out. Sodium periodate targets any saccharide/polysaccharide leading to the oxidation of these residues. The spent *A. fumigatus* media (100 ml) were pre-treated with 20-mM sodium periodate (Cat No. S1147, Sigma) solution prepared in an oxidation buffer (50-mM sodium acetate and 50-mM acetic acid, pH-4.5). Since sodium periodate is light sensitive, the preparation of the solution and pre-treatment of *A. fumigatus* spent medium were performed in the dark at 28 °C and 150 rpm. To stop the activity of sodium periodate, the sample was exposed to light for 15 min to remove the remaining periodate in the system.

The methanol: chloroform extraction was performed by mixing an equal volume of methanol: chloroform mixture (1:1) with the *A. fumigatus* spent medium for 1 h at 28 °C and 150 rpm to identify any polar molecules responsible for the harvesting process. The aqueous fraction was separated from the mixture by a separating funnel and the organic fraction was discarded. Washed *A. fumigatus* pellets suspended in this aqueous fraction were then subjected to harvesting of *C. pyrenoidosa* cells. A similar experiment was done using the hexane-extracted aqueous fraction (extracted following the similar protocol used for Methanol: Chloroform aqueous extract) to identify the presence of any non-polar molecule which aided in the harvesting process. The *A. fumigatus* spent medium was analyzed using high-resolution liquid chromatography–mass spectrometry (HR-LC–MS).

### Determination of mediating molecule

Based on the HR-LC–MS analysis and preceding *A. fumigatus* spent medium treatments, glucose and N-acetyl glucosamine (GlcNAc) were suspected to play the most important role in harvesting process. *C. pyrenoidosa* biomass was exposed to 100-mM glucose and GlcNAc, respectively, by incubation for 2 h at 38 °C and 100 rpm, followed by removal of residual glucose and GlcNAc by centrifugation (4000*g* for 10 min) and resuspension of biomass in fresh BG11. *C. pyrenoidosa* after glucose/GlcNAc incubation was then subjected to harvesting with washed *A. fumigatus* pellets (without spent medium) for 5 h as described in “*C. pyrenoidosa* and *A. fumigatus* harvesting experiments” section. Another set of *C. pyrenoidosa* suspension was also incubated under the same conditions without supplementation of glucose/GlcNAc followed by its harvesting with washed *A. fumigatus* pellets. To analyze the concentration of glucose and GlcNAc at different stages of experiment, high-performance liquid chromatography (HPLC) was performed at three levels: (i) supernatant of *C. pyrenoidosa* suspension immediately after glucose or GlcNAc addition (initial); (ii) supernatant of *C. pyrenoidosa* suspension after 2 h of incubation with glucose or GlcNAc and (iii) supernatant of respective sets at the end of harvesting for 5 h. The results were compared with HPLC of glucose and GlcNAc standard as a reference. HR-LC–MS of the supernatants from all the set after harvesting was also done to confirm the result of HPLC.

GlcNAc-incubated *C. pyrenoidosa* biomass was also subjected to Fourier Transform Infra-Red Spectroscopy (FTIR) and was compared to FTIR spectra of *C. pyrenoidosa* biomass during harvesting (after 2 h) as well as normal *C. pyrenoidosa*. The FTIR of GlcNAc powder was also performed to correlate the presence of similar peaks on the incubated *C. pyrenoidosa* biomass. To further confirm the role of GlcNAc and *A. fumigatus* spent medium in the harvesting process, *C. pyrenoidosa* cells incubated with GlcNAc (100 mM) and *A. fumigatus* spent medium was observed under a fluorescent microscope and high-resolution transmission electron microscope (HR-TEM, Sect. 2.7.5) and was compared with normal *C. pyrenoidosa* cells. For fluorescent microscopy, the cells were stained with Concanavalin A (Con A) conjugated with Alexa Fluor^®^ 594 (Cat No. C11253, ThermoFisher Scientific) to detect the presence of GlcNAc on the *C. pyrenoidosa* cell surface [62], followed by viewing of cells under a fluorescent microscope (Nikon Eclipse Ti-U). Con A is a lectin which binds d-glucose, d-fructose, d-mannose, N-acetyl-d-glucosamine and related monosaccharides [63]. Since Con A does not have fluorescence, it is tagged with a fluorescent dye Alexa Fluor 594 (Excitation/Emission: 590/617 nm). To 100 µl of *C. pyrenoidosa* cells, 0.1 µl of the dye was added and kept in the dark for 5 min at room temperature. The stained cells were then observed under the microscope.

### Analytical techniques

#### Sample preparation for high-performance liquid chromatography (HPLC) and high-resolution liquid chromatography–mass spectrometry (HR-LC–MS)

Samples were made salt free by passing through polymeric cartridge Strata-X-CW (Cat No. 8B-S035-JEG, Phenomenex). The cartridge was first conditioned by adding 10-ml methanol followed by 10-ml distilled water. The flow-through was discarded, and then 10 ml of the sample was added to the cartridge. The flow-through from the cartridge was collected, tested for harvesting activity and then analyzed using HPLC and HR-LC–MS.

#### HPLC

Standards for glucose (Cat No. 47829) and GlcNAc (Cat No. PHR1432-1G) were purchased from Sigma. Standards were dissolved in HPLC grade water (Cat No. AS077-1L, Hi-Media). HPLC was performed according to manufacturer’s protocol using Agilent 1260 series machine with Agilent Hi-Plex H column and RI detector. The mobile phase was 0.005-M H_2_SO_4_ (Cat No. 5438270100, Sigma) at a flow rate of 0.6 ml min^−1^. The column temperature was kept at 60 °C and the run time was 20 min. All samples were degassed prior to analysis.

#### HR-LC–MS

HR-LC–MS was done to identify the nature of the compounds present in the *A. fumigatus* spent medium. The *A. fumigatus* spent medium is a mixture of various types of compounds like proteins, carbohydrates, organic acids, sugars and *A. fumigatus* metabolites. Hence, HR-LC–MS was done for these compounds. The aliquot was subjected to HR-LC–MS analysis (ACCUCORE RP-MS), and was outsourced to Sophisticated Analytical Instrument Facility (SAIF), Indian Institute of Technology (IIT), Mumbai. The column used was ZORBAX ECLIPSE C-18 (Agilent Technologies). The sample was run isocratically for 30 min using acetonitrile (95%) as a solvent. The compounds were analyzed using Quadrupole—Time of Flight mass spectrometer (Q-TOF MS; Agilent iFunnel G6550A) giving the probable hits from the database library provided by the manufacturer.

#### Scanning electron microscopy (SEM)

SEM analysis of (i) normal *C. pyrenoidosa* cells, (ii) *C. pyrenoidosa* cells incubated with *A. fumigatus* spent medium, (iii) GlcNAc-incubated *C. pyrenoidosa* and (iv) washed *A. fumigatus* pellets were done using a previously described protocol [61]. The samples for SEM analysis were first washed with PBS and then fixed with 1% glutaraldehyde for 4 h at room temperature. The samples were centrifuged and the fixative was discarded. Samples were then lyophilized (Allied Frost FD3) for SEM analysis using a ZEISS EVO 50 instrument under the following analytical condition: EHT = 20.00 kV, WD = 9.5 mm, Signal A = SE1.

#### High-resolution Transmission electron microscopy (HR-TEM)

The *C. pyrenoidosa* cells ((i) normal *C. pyrenoidosa* cells, (ii) *C. pyrenoidosa* cells incubated with *A. fumigatus* spent medium, (iii) GlcNAc-incubated *C. pyrenoidosa* and (iv) washed *A. fumigatus* pellets) were centrifuged at 4000*g* for 10 min and prepared for viewing as outlined by Gola et al. [64]. The samples were examined by high-resolution transmission electron microscope (Tecnai G2 20) operated at 200 kV.

#### Atomic force microscopy (AFM)

To confirm the observations of TEM analysis, AFM studies of the *C. pyrenoidosa* cells (normal cells and *A. fumigatus* spent medium-incubated *C. pyrenoidosa* cells) were done. *C. pyrenoidosa* cells were centrifuged and suspended in 50-mM citrate–phosphate buffer (pH 3) for conditioning. The buffer was removed by centrifugation, and the pellets were kept on glass cover slip for 30 min followed by washing with de-ionized water and then air drying. AFM micrographs were obtained using Bruker INNOVAA2 Sys instrument in tapping mode.

#### Fourier transform infrared spectroscopy (FTIR)

For FTIR measurements, the samples were first washed with phosphate buffer saline (PBS) and then lyophilized. The lyophilized powder was then used for FTIR analysis using a Nicolet Is50 (Thermo Scientific) instrument.

#### Statistical analysis

All experiments were performed in triplicates and results were represented as mean ± S.D wherever applicable. Graphs were drawn using Microsoft Excel^®^ (Part of Microsoft Office 2013 package). Significance test has been done using one-way ANOVA (*p* < 0.05). *t* Test (two-tailed) was also performed to check the significance between two data sets.

## Additional files

**Additional file 1: Figure S1.** Harvesting kinetics of *C. pyrenoidosa* with *A. fumigatus* pellets after incubation with glucose and GlcNAc showing significant difference in process kinetics (*p* < 0.05) up to 4 h. Non-significant difference (*p* < 0.05) was observed between harvesting kinetics of control and GlcNAc.

**Additional file 2: Table S1.** HR-LC–MS analysis of supernatants after harvesting of algal–fungal mixtures using algae with different pre-incubations.

**Additional file 3: Figure S2.** Representative brightfield (left) and fluorescent (right) micrographs of SYTOX Green stained *C. pyrenoidosa* cells incubatedwith *A. fumigatus* spent medium.

**Additional file 4: Figure S3.** Harvesting efficiency of *C. pyrenoidosa* cells with *A. fumigatus* when incubated with different sugars.

## Data Availability

Data supporting the results of the article are included within this manuscript and additional information.

## Abbreviations

AF: *Aspergillus fumigatus*
AFM: atomic force microscopy
CP: *Chlorella pyrenoidosa*
FTIR: Fourier transform infrared spectroscopy
GlcNAc: *N*-acetyl glucosamine
HPLC: high-performance liquid chromatography
HR-LC–MS: high-resolution liquid chromatography–mass spectrometry
OD: optical density
PDA: potato dextrose agar
PDB: potato dextrose broth
SEM: scanning electron microscopy
TEM: transmission electron microscopy
