# Supplementary material for: Potential role of N-acetyl glucosamine in Aspergillus fumigatus-assisted Chlorella pyrenoidosa harvesting
Source: Biotechnol Biofuels. 2019 Jul 10;12:178. doi: 10.1186/s13068-019-1519-3 (PMC6617575; doi:10.1186/s13068-019-1519-3)
Supplement: Supplementary file 1 — Additional file 1: Figure S1. Harvesting kinetics of C. pyrenoidosa with A. fumigatus pellets after incubation with glucose and GlcNAc showing significant difference in process kinetics (p < 0.05) up to 4 h. Non-significant difference (p < 0.05) was observed between harvesting kinetics of control and GlcNAc. [file 13068_2019_1519_MOESM1_ESM.docx]

**Additional file 1: Figure S1: Harvesting kinetics of *C.pyrenoidosa* with *A.fumigatus* pellets after incubation with glucose and GlcNAc showing significant difference in process kinetics (p<0.05) up to 4 h. Non-significant difference (p<0.05) was observed between harvesting kinetics of control and GlcNAc**
