# Supplementary material for: Potential role of N-acetyl glucosamine in Aspergillus fumigatus-assisted Chlorella pyrenoidosa harvesting
Source: Biotechnol Biofuels. 2019 Jul 10;12:178. doi: 10.1186/s13068-019-1519-3 (PMC6617575; doi:10.1186/s13068-019-1519-3)
Supplement: Supplementary file 2 — Additional file 2: Table S1. HR-LC–MS analysis of supernatants after harvesting of algal–fungal mixtures using algae with different pre-incubations. [file 13068_2019_1519_MOESM2_ESM.docx]

**Additional file 2: Table S1: HR-LC-MS analysis of supernatants after harvesting of algal-fungal mixtures using algae with different pre-incubations**

| **Category of compound** | **Algae without Glucose/GlcNAc pre-incubation** | **Algae with Glucose pre-incubation** | **Algae with GlcNAc pre-incubation** |
| --- | --- | --- | --- |
| Sugars (Monosaccharides, Polysaccharides) | ND | Lactose, D-Glucose | ND |
| Sugar derivatives (Sugar alcohols, ketones, aldehydes, esters) | Retinylphosphate mannose | N-Acetylglucosamine, Mannitol, Neuraminic acid | N-Acetylglucosamine, Retinylphosphate mannose |
| Organic acids and its derivatives | Ethyl Oxalacetate, Triparanol | Gluconic acid, Propranolol glucuronide, Fumarylacetoacetic acid | L-2-Aminoadipic acid, Fumarylacetoacetic acid |
| Lipid, Fatty acids and its methyl esters | 10-keto tridecanoic acid, 9-Keto heptadecylic acid, Methyl N-(amethylbutyryl) glycine, N-Acrylylglycine methyl ester, 9-amino-nonanoic acid | Propionylglycine methyl ester | 3-Hydroxydodecanedioic acid, Docosanedioic acid, 3beta,6alpha,7alpha-  Trihydroxy-5beta-cholan-24-  oic Acid |
| Nitrates, Nitrites, amines and other nitrogen derivatives | p-Chlorobenzenesulfonylurea | Nitroglycerine | 4-[[5-(acetylamino)-1-methyl-  1H-indol-3-yl]methyl]-3-  methoxy-N-[(2-  methylphenyl)sulfonyl]-  Benzamide |
| Protein metabolites (amino acid sequences) | Urocanic acid, Lys Gly Pro | Pro His, Gln Asp Ser | Arg Gln Arg, Ile Trp Thr |
| Vitamins and its derivatives | Choline, Methacholine | Succinylmonocholine, Pantetheine | 1alpha,25-dihydroxy-3-deoxy- 3-thiavitamin D3 3-oxide / 1alpha,25-dihydroxy-3-deoxy- 3-thiacholecalcif , Pantetheine |
| Nucleic acids and its derivatives | Tetrahydropteridine | ND | ND |
| Other metabolites and compounds | Pyrocatechol sulfate, Norcotinine, Ethosuximide M5, Zolpidem Metabolite II | Chenodeoxycholic acid sulfate, Betamethasone, protoporphyrin IX, Idebenone Metabolite, Peridinin | Hydrocortisone  Phosphate, 4-Amino-4-deoxychorismic  Acid, Chenodeoxycholic acid sulfate, Betamethasone, protoporphyrin IX, Peridinin, Presqualene diphosphate |
