# Supplementary material for: Potential role of N-acetyl glucosamine in Aspergillus fumigatus-assisted Chlorella pyrenoidosa harvesting
Source: Biotechnol Biofuels. 2019 Jul 10;12:178. doi: 10.1186/s13068-019-1519-3 (PMC6617575; doi:10.1186/s13068-019-1519-3)
Supplement: Supplementary file 3 — Additional file 3: Figure S2. Representative brightfield (left) and fluorescent (right) micrographs of SYTOX Green stained C. pyrenoidosa cells incubatedwith A. fumigatus spent medium. [file 13068_2019_1519_MOESM3_ESM.docx]

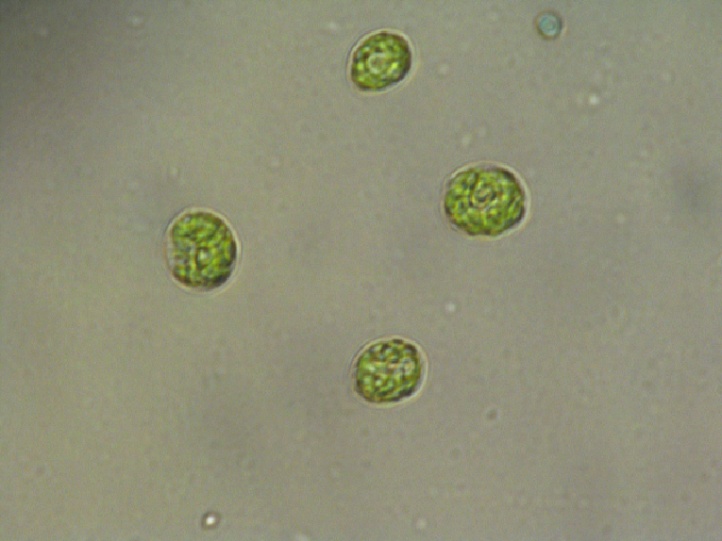

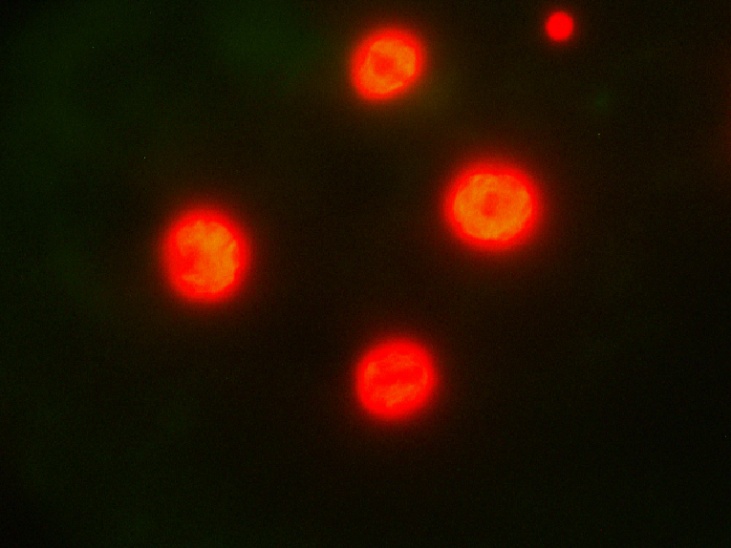


**Additional file 3: Figure S2: Representative brightfield (left) and fluorescent (right) micrographs of SYTOX Green stained *C.pyrenoidosa* cells incubated with *A.fumigatus* spent medium**
