# Supplementary material for: Potential role of N-acetyl glucosamine in Aspergillus fumigatus-assisted Chlorella pyrenoidosa harvesting
Source: Biotechnol Biofuels. 2019 Jul 10;12:178. doi: 10.1186/s13068-019-1519-3 (PMC6617575; doi:10.1186/s13068-019-1519-3)
Supplement: Supplementary file 4 — Additional file 4: Figure S3. Harvesting efficiency of C. pyrenoidosa cells with A. fumigatus when incubated with different sugars. [file 13068_2019_1519_MOESM4_ESM.docx]

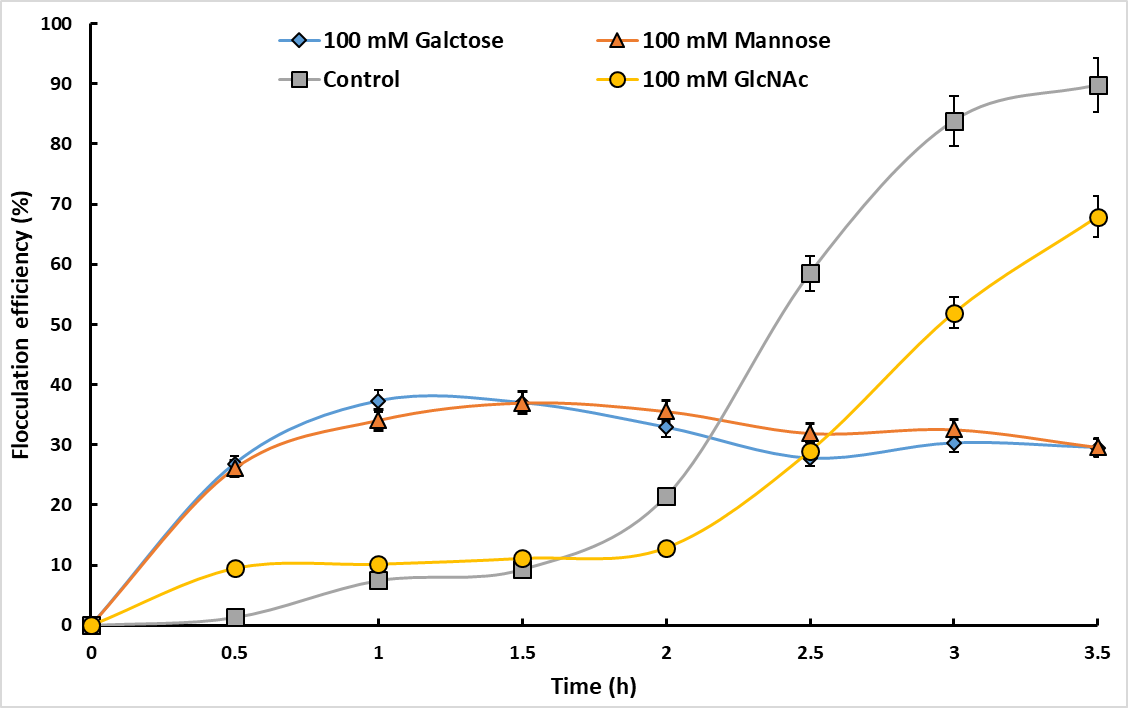


**Additional file 4: Figure S3: Harvesting efficiency of *C.pyrenoidosa* cells with *A.fumigatus*  when incubated with different sugars.**
